# A novel traction method for underwater colorectal endoscopic submucosal dissection: reopenable clip-over-the-line traction

**DOI:** 10.1055/a-2644-7901

**Published:** 2025-07-29

**Authors:** Tatsuma Nomura, Takanobu Mitani, Yuto Ikadai, Takanori Takenaka, Hiroaki Kumazawa, Yoshiaki Isono, Katsumi Mukai

**Affiliations:** 1 Department of Gastroenterology, Suzuka General Hospital, Suzuka, Mie, Japan; 2 Department of Endoscopy Center, Suzuka General Hospital, Suzuka, Mie, Japan

Recently, the usefulness of colorectal endoscopic submucosal dissection (ESD) with saline immersion has been reported in many studies. Underwater ESD improves scope manipulation, magnifies the field of view, and provides hemostasis 1 . Underwater ESD creates buoyancy, and the dissection collapse of the cavity makes applying strong traction to the lesion difficult. Therefore, we propose a reopenable clip-over-the-line traction (ROLT) technique in which the traction strength can be adjusted during underwater ESD.

The patient was a male in his 50 s with colorectal tumor approximately 40 mm in size surrounding the appendiceal orifice ( Fig. 1 , Video 1 ). The tumor was resected en bloc using a gas-free saline immersion system 2 . The tumor was resected using a calibrated, small-caliber tip, transparent hood (CAST hood; TOP, Tokyo, Japan) with a tapered tip, which allowed visualization of the border between the tumor and normal mucosa of the appendix. First, an incision was made in the normal mucosa of the appendix. A submucosal flap was created after making an incision around the entire circumference and dissecting the submucosa. Traction was then applied using ROLT with a reopenable clip tooth hole and line. First, a clip with line was placed on the mucosa at the lesion margin. Subsequently, the reopenable-clip over the line method was used, with a clip and line threaded through the hole in one side of the teeth inserted through the accessory channel 3 4 . Thereafter, three additional clips were placed on the normal mucosa in the same manner. Finally, one clip was placed on the normal mucosa on the anal side. The lesion was dissected to the appropriate submucosal layer with strong traction to the anal side. The tumor was completely resected, and the lines were cut using M-LCT 5 . The patient was discharged without any adverse events, including post-ESD electrocoagulation syndrome.

**Fig. 1:**
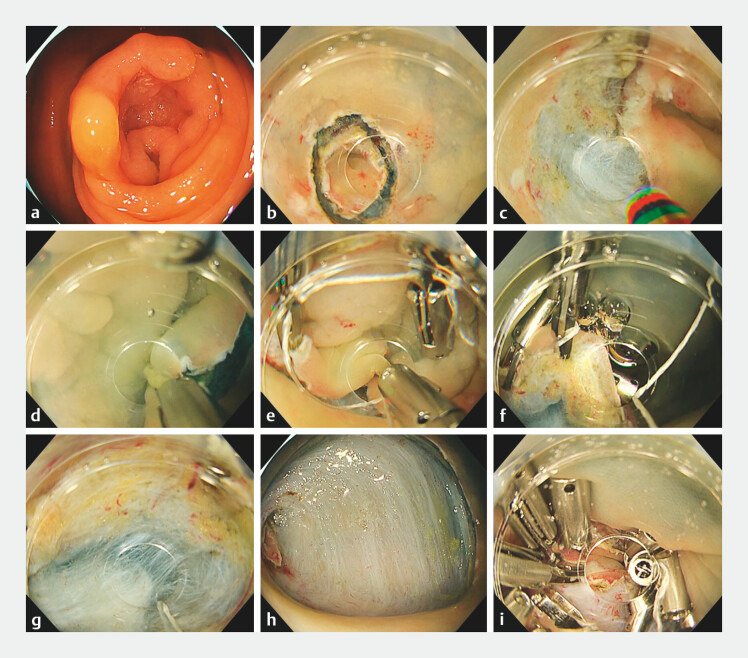
ESD of a cecal tumor with saline-immersion using reopenable clip-over-the-line traction (ROLT). **a** Sessile serrated lesion around the appendiceal orifice measuring 40 mm in size. **b** After an incision in normal mucosa on the appendiceal side. **c, d** After the entire mucosal incision and dissection of the submucosa, a clip with line and clip was placed on the normal mucosa on the side of the lesion. **e** A reopenable clip with a line through the tooth hole on one side was placed on the normal mucosa on the lesion side. **f** Finally, a reopenable clip through the tooth hole on one side was placed on the contralateral normal mucosa on the anal side to obtain strong traction (reopenable clip over-the-line traction: ROLT). **g** As the scope approached the submucosa, stronger traction was applied to the submucosa using the pulley principle. **h** Mucosal defect after complete resection, with an ESD procedure time of 40 min. The resected specimen was 50 mm in size, and the tumor was identified as a sessile serrated lesion. **i** The normal mucosa of the Appendix and the normal mucosa of the cecum were fixed using the reopenable clip-over-the-line method (ROLM) twice so that the appendiceal orifice was maintained. The remaining mucosal defect was then completely closed using a third ROLM.

Endoscopic submucosal dissection for a sessile serrated lesion near the appendix using reopenable clip-over-the-line traction.Video 1

Endoscopy_UCTN_Code_TTT_1AQ_2AD_3AD

## Notes

Endoscopy E-Videos https://eref.thieme.de/e-videos
*E-Videos* is an open access online section of the journal *Endoscopy* , reporting on interesting cases and new techniques in gastroenterological endoscopy. All papers include a high-quality video and are published with a Creative Commons CC-BY license. Endoscopy E-Videos qualify for HINARI discounts and waivers and eligibility is automatically checked during the submission process. We grant 100% waivers to articles whose corresponding authors are based in Group A countries and 50% waivers to those who are based in Group B countries as classified by Research4Life (see: https://www.research4life.org/access/eligibility/ ). This section has its own submission website at https://mc.manuscriptcentral.com/e-videos .
